# Alterations in the Morphology of the Testis and Epididymis Caused by the Consumption of Hyperlipidic Diets in Wistar Rats

**DOI:** 10.3390/life15060959

**Published:** 2025-06-14

**Authors:** Rosario Tarragó Castellanos, Maria Mendoza Delgado, Lorena Ruiz Valderrama, Isabel Arrieta Cruz, Edith Cortés Barberena, Adriana Morales Otal, Ernesto Rodríguez Tobón, Ahiezer Rodríguez Tobón, Javier Olvera Ramírez, Edith Arenas Ríos

**Affiliations:** 1 Departamento de Biología de la Reproducción, División de Ciencias Biológicas y de la Salud, Universidad Autónoma Metropolitana, Ciudad de México 09340, Mexico; mrtc@xanum.uam.mx (R.T.C.);; 2 Maestría en Biología de la Reproducción Animal, División de Ciencias Biológicas y de la Salud, Universidad Autónoma Metropolitana, Ciudad de México 09340, Mexico; 3 Departamento de Investigación Básica, Instituto Nacional de Geriatría, Ciudad de México 1200, Mexico; 4 Departamento de Ciencias de la Salud, División de Ciencias Biológicas y de la Salud, Universidad Autónoma Metropolitana, Ciudad de México 09340, Mexico; 5 Departamento de Biología, División de Ciencias Biológicas y de la Salud, Universidad Autónoma Metropolitana, Ciudad de México 09340, Mexico

**Keywords:** histomorphometry, sperm quality, testis, epididymis, overweight, obesity

## Abstract

Obesity is considered a metabolic disease, in which leptin is used as an indicator of energy in the body. This hormone, in turn, is related to the neuroendocrine regulation of the reproductive axis. However, leptin excess secretion due to obesity can have a negative effect on reproduction. Overweight and obesity were induced through high-calorie diets. Lee and gonadosomatic indices were determined to characterize the model and degree of reproductive development in the testis and epididymis. Sperm quality was analyzed using spermatobioscopy. Morphometry was analyzed through histological analysis. The changes described affect testicular function in hormone and sperm production. Exposure of 3-month-old male rats to diets with different fat contents (10% and 60%) induced both overweight and obesity. The animals showed morphological alterations, both testicular and epididymal, the latter being more sensitive to dietary changes by modifying the epididymal index, morphometric parameters (in both organs), and a decrease in cilia length. These changes induced a reduction in sperm viability, as well as an increase in malformed spermatozoa. In conclusion, both overweight and obesity have effects on male reproduction by modifying the morphology and physiology of reproductive organs.

## 1. Introduction

Obesity has been considered a chronic metabolic disease of multifactorial origin in men [1]. It is expected that by 2025, 18% will suffer from it, which is why it is already considered a global health problem [2]. The WHO defines obesity as an increase in body weight induced by a positive energy balance, which generates an accumulation of adipose tissue [3].

Overweight and obesity are correlated with an increased body mass index (BMI) and male infertility. Individuals with a BMI greater than 30 have hypogonadism and, consequently, impaired semen quality [4,5].

In men, the testis has a dual function, being responsible for the production of testosterone as well as sperm cells. Testosterone synthesis is carried out in Leydig cells by stimulation of pituitary hormones, which in turn are regulated by hypothalamic hormonal secretions [1,6,7].

Inside each testicle are the seminiferous tubules, where spermatogenesis takes place. These seminiferous tubules empty into the rete testis, which are anastomosing canals located within the testicular medulla. The epithelium is involved in the endocytosis of proteins and molecules from the lumen of the seminiferous tubules. The rete testis is continuous with the efferent ducts, which communicate with the epididymis [8,9].

The epididymis is a highly coiled duct that varies in length depending on the species; in rats, it can measure up to 3 m, and in humans, 3 to 6 m [10]. It is an androgen-dependent organ, so it requires testosterone, which is metabolized in the main cells to dihydrotestosterone, necessary for its proper functioning [8,11,12].

The newly formed spermatozoon, released into the lumen of the seminiferous tubules of the testicle, is still an immature spermatozoon and to acquire the fertilizing capacity, it needs to undergo structural, morphological, and biochemical changes in the epididymis to acquire its capacity for movement and produce the fusion of its genetic material with that of the female gamete [13].

The epididymis is divided into segments, and each convoluted duct is separated by a septum [14,15]. One of the most important functions of the epididymis is to contribute to the formation of an ideal luminal microenvironment, which is essential for sperm to reach maturity in the cauda region [15].

In several obese men, this condition has been associated with elevated levels of estradiol (E2), leptin, decreased testosterone levels, and gonadotropic hormones. The conversion of androgens to estrogens takes place in adipose tissue; this conversion is greater, so negative signaling at the hypothalamic and pituitary level results in an alteration in testosterone synthesis [8]. Generally, in mammals, androgen/estrogen balance is necessary for sexual activity and for reproduction to take place.

Leptin participates in neuroendocrine regulation, a hormone produced and secreted by adipocytes and involved in sexual maturation and reproduction by acting as an energy signal in the body [6]. The function of leptin is to stimulate the synthesis and secretion of kisspeptins that regulate the secretion and release of hypothalamic gonadotropin-releasing hormone (GnRH), which will stimulate the secretion of follicle-stimulating hormone (FSH) and luteinizing hormone (LH) at the pituitary level.

Therefore, the increase in body mass due to an increase in adipose tissue will result in alterations in the regulation of the hypothalamus–pituitary–testicle axis, and therefore, in male fertility. One of the changes observed in conditions of obesity is an increase in scrotal temperature generated by the increase in adipose tissue; this change will translate into reproductive alterations by affecting sperm quality, which is observed by a decrease in sperm motility and concentration, the presence of morphological abnormalities, sperm DNA fragmentation, in addition to damage to Leydig cells, hypogonadism, and a decrease in epididymal size [9]. It has been reported that obese men of reproductive age frequently present with low libido and reduced fertility.

Some of the aspects mentioned above have already been evaluated by us, and we have found that overweight and obesity can affect epididymal sperm maturation. However, the effect of these conditions has not been analyzed on testicular and epididymal morphology and histology in the Wistar rat strain. The use of rodents, such as the Wistar rat strain, in research is an experimental tool for studying various physiological and pathological processes, given that they have certain characteristics similar to humans. They also reproduce rapidly and continuously, as their life cycle is short. Wistar rats are used as models in studies of overweight and obesity by modifying their diet [16]. The correlation of obesity with low fertility in humans has been analyzed by several authors; overweight conditions have been less studied, even though it is not less important than obesity. The study aims to show a biological model used to analyze the effect of these two conditions, overweight and obesity, on testicular and epididymal morphology and sperm quality under controlled feeding conditions.

In general, human studies have examined obesity as a cause of infertility. The present research studied the histological changes in the testes and epididymides induced by two high-fat diets. When consumed over a relatively short period (one month), these diets alter epididymal parameters by inducing overweight and obesity. This study provides new knowledge about the changes these conditions induce at the tissue level, enabling us to develop therapeutic strategies related to obesity and infertility.

## 2. Results

### 2.1. Lee Index

Significant differences were observed in the Lee index (*p* < 0.001); the rats in the control group had a Lee index lower than 3, unlike the rats that consumed the diets with 10% and 60% fat; hence, the observed values allowed the former to be considered overweight and the latter obese (Table 1).

### 2.2. Scrotal Fat

The increase in scrotal fat was significantly higher (*p* < 0.001) in the overweight and obese group than in the control group. The overweight group showed a twofold increase compared to the control group, and the obesity group was four times greater than the control and twice as large as the overweight group (Table 1).

### 2.3. Gonadosomatic Index (GSI) and Epididymal Somatic Index (ESI)

In the GSI, no significant differences were observed between the control group, the overweight group, and the obese group. The ESI of the obesity group was significantly lower (*p* < 0.001) than that of the control group and the overweight group (Table 1).

### 2.4. Histological Analysis

#### Testicle

**Figure 1:**
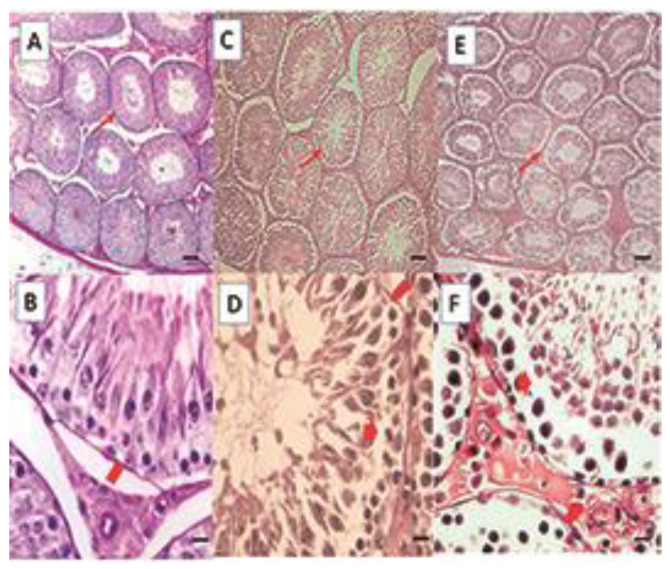
Histological sections of the testis. Control (**A**,**B**), DIO (**C**,**D**), DIOb (**E**,**F**). The seminiferous tubules of the overweight and obese animals show vacuolization, which generates a displacement of the seminiferous epithelium towards the tubule lumen in some tubules, folding of the basement membrane is observed, and the shape of the tubule is irregular when compared with the control group. Hematoxylin–Eosin technique. Thin arrow: seminiferous tubule; Thick arrow: vacuolization; Arrowhead: basement membrane. Bar 100 µm micrograph (**A**,**C**,**E**); Bar 20 µm micrograph (**B**,**D**,**F**).

In the control group, normal tubules are observed. Sertoli cells are located at the base of the tubule with a stratified germline epithelium with linear progression of spermatogenesis from the basement membrane to the lumen of the tubule, in which the presence of spermatozoa is observed. Eosinophilic Leydig cells are observed in the interstitium (Figure 1A,B).

In the animals with overweight (Figure 1C,D) and obesity (Figure 1E,F), the alterations are similar; in group DIO, these can be observed in 40% of the tubules of each animal, and in group DIOb, in 96–100% of the animals. The basal membrane is vacuolized and folded in some areas; the wall of the tubules seems thinner. The observed vacuolization produces a displacement of the seminiferous epithelium. Spermatozoids can be observed in the lumen; however, there are fewer than in the control group. This can be due not only to a disorganization of the seminiferous epithelium but also to a reduction of late spermatids, which is more frequent in the diet that induces obesity. In the histomorphometric analysis, an increase in the seminiferous tubule diameter is observed in both fat-rich diets; however, only the obese group was significantly different (*p* < 0.001) from the overweight group and the control group (Table 2).

Significant differences (*p* < 0.001) were found in seminiferous epithelial height between all groups. In the control group, epithelial height was greater than in the overweight group but less than in the obesity group. The increase in the obesity group could be due to vacuolization and epithelial sloughing, since measurements were made from the membrane to the tubule lumen (Table 2).

- Epididymis

**Figure 2:**
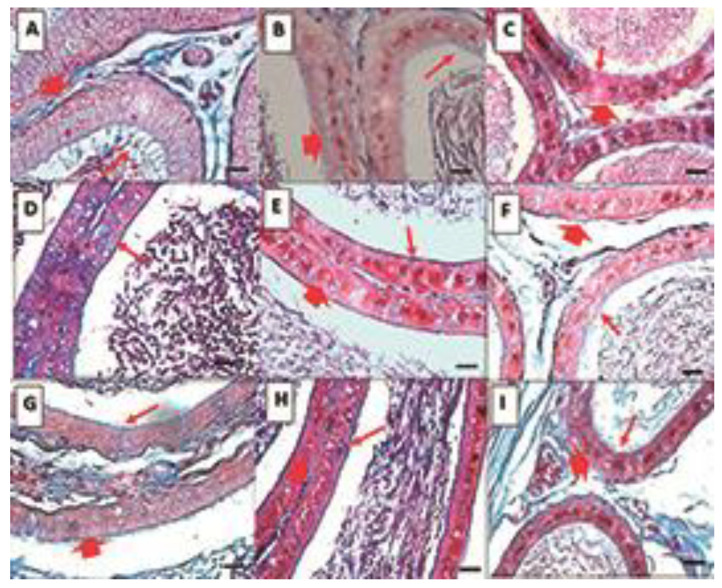
Histological sections of the epididymis. Control (**A**–**C**), DIO (**D**–**F**), DIOb (**G**–**I**). Sections of the three regions of the epididymis, *caput* (**A**,**D**,**G**), *corpus* (**B**,**E**,**H**), and *cauda* (**C**,**F**,**I**). In the three sections of the epididymis in the control group, it is observed that along the epididymis, there is a decrease in the size of the epithelium, in the *caput* region, with an abundance of cilia. Spermatozoa are observed in the center of the tubule. Both the overweight and obesity groups show a decrease in the above-mentioned parameters, as well as an increase in vesicles in the epithelium of the treated animals. Changes are observed in the number and length of cilia, which decrease significantly in the *corpus* and *cauda.* In the obese animals, the changes are more drastic in terms of size and number in the *corpus* and *cauda* regions. Masson’s trichrome. Thin arrow: cilia; Thick arrow: epithelium. Bar 20 µm.

Histological analysis of the epididymis is presented for each of the epididymal regions (Figure 2).

*Caput*: In the control group, round ducts are observed, the lumen is reduced with the presence of spermatozoa in the center, and pseudostratified epithelium (basal and main cells) has the presence of abundant, long, and thick cilia. In the overweight group, the shape of the duct is oval, and the lumen is larger, probably due to the decrease of spermatozoa; the epithelium is thinner and there are vacuoles, the cilia are scarce, and of smaller size and thickness. Finally, in the obesity group, the shape of the ducts is irregular, the lumen is perceived as larger, probably due to the decrease in spermatozoa, which are dispersed. The pseudostratified epithelium has an abundance of larger vacuoles and scarce and small cilia.

*Corpus*: In the control group, unlike the head region, the shape of the duct is oval, the pseudostratified epithelium is thinner with fewer cilia, which are also thinner, and the spermatozoa are observed grouped in the lumen. In the obesity group, the shape and thickness of the epithelium are similar to the control; large vacuoles are observed in the epithelium, and the spermatozoa are not grouped. In the obesity group, the changes are like those described in the caput region.

*Cauda*: The shape of the ducts in the control group is slightly oval, the epithelium is thinner than in the previous regions, with almost no presence of cilia, which are observed to be small, and the spermatozoa are grouped in the lumen of the tubule. In the overweight group, the shape of the ducts is more irregular, the epithelium is like that of the control group, but unlike the control group, there are vacuoles, and the spermatozoa are not aggregated. The obesity group is like the overweight group, but with a greater abundance of vacuoles.

### 2.5. Histomorphometric Analysis

- Duct Area

In the control group, the duct area is smaller in the *caput* region compared to the other two areas of the epididymis, *corpus*, and *cauda*.

When comparing the *caput* region between the three groups, we observed a significant increase in both overweight and obesity, with the increase being greater in the latter (*p* < 0.001). The opposite occurred in the *corpus* region, with a significant decrease in both overweight and obesity, with the area being smaller in the latter (*p* < 0.001) (Table 3).

- Percentage of duct lumen

In the control group, the duct lumen was smaller in the *caput* region compared to the other two regions. In the overweight and obesity groups, a higher percentage of the duct corresponded to the lumen of these compared to the control group (*p* < 0.001). However, in the *corpus*, only the overweight group showed differences (*p* < 0.001) compared to the control group. In the cauda group, both were significantly smaller (*p* < 0.001) than the control group, but obesity was greater than overweight (Table 3).

- Percentage of Ductal Area Occupied by Sperm

In the control group, the percentage of space occupied by sperm in the ductal lumen was constant in all three epididymal areas. A significant increase (*p* < 0.001) was observed in the *caput* area compared to the control group in both overweight and obesity groups, with a greater increase in the latter.

In the overweight group, a slight decrease was observed in the corpus area compared to the other two (*p* < 0.001), and in the obesity group, a greater space was occupied by spermatozoa (Table 3).

- Epithelial Height

In the *caput* region, we observed a significant decrease (*p* < 0.001) in the overweight and obesity group, which was the area where the greatest change occurred. The *corpus* region showed the same pattern of decreased height as in the anterior region (*p* < 0.001). Finally, in the *cauda* region, only the overweight group showed a significant decrease (*p* < 0.001) compared to the other two groups (Table 3).

- Cilia length

**Figure 3:**
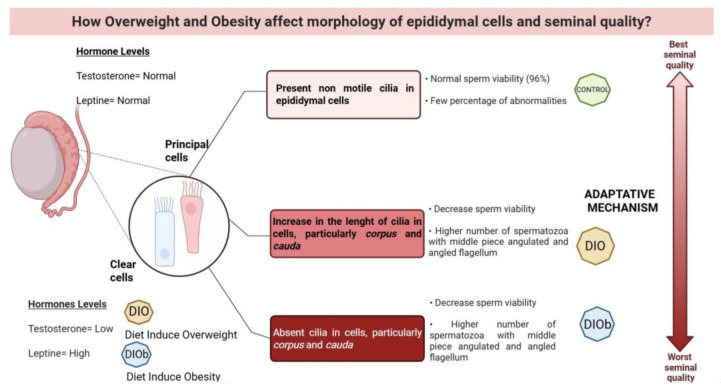
Changes that occur in the clear and principal cells of the epididymis, as well as the effect on sperm viability and morphology due to overweight and obesity in Wistar rats. A low concentration of testosterone and high leptin due to the increase in adipose tissue in obesity generate changes in the length of the cilia in the principal and clear cells of the epididymis, particularly in the corpus and cauda parts, which is proposed to be due to an adaptive mechanism. Although the cells try to compensate for the degenerative effect on the cell by lengthening the cilia, under conditions of overweight, in the end, obesity causes the cilia to be lost in these cells, and seminal quality is affected, decreasing viability and increasing abnormalities in the midpiece and flagellum of the sperm, created in BioRender.com, 2025.

In the control group, cilia length was observed to be greater in the *caput* region, decreased in the *corpus* region, and even shorter in the *cauda* region. In overweight animals, a decrease in the *caput* area and a significant increase (*p* < 0.001) in the length of cilia located in the *corpus* and *cauda* were observed. However, in the obesity group, a decrease (*p* < 0.001) was observed in all three areas of the epididymis compared to the other two groups (Table 4, Figure 3).

The following results were obtained: vitality in the control group was 96%, in the overweight group it decreased significantly (*p* < 0.001) to 70% and 32% in the obesity group. The presence of an amorphous head was significantly higher (*p* < 0.001) in the obesity group compared to the control group. The presence of angulated (*p* < 0.001), asymmetric (*p* < 0.001), as well as angulated (*p* < 0.001) and coiled flagellum (*p* < 0.001) was significantly different among the three groups (Table 5).

## 3. Discussion

In recent years, obesity has been on the rise, favoring pathologies including male infertility. Studies related to the effects of obesity on male fertility have mainly focused on sperm parameters [17]. However, the present study aims to explore etiology and to establish effective treatments for these pathologies.

In the present study, administration of a diet containing 10 and 60% saturated fatty acids for 4 weeks was sufficient to induce this response. In laboratory animals such as rats, the Lee index is considered an accurate way to determine overweight and obesity induced by hyperlipidemic or hyperglycemic diets [17,18,19], reporting that a value of 0.294 g is indicative of obesity [18]. Vigueras-Villaseñor et al. (2011) [20] agree that a value of 0.3 in rats is indicative of obesity, so our data (0.29 and 0.32) can be considered as indicators of overweight and obesity.

Human studies have linked weight gain in men to reduced testosterone levels, as well as decreased sperm quality and fertility, compared to men of normal weight [21]; reduced testosterone levels may be due in part to aromatization of this hormone in peripheral tissue, inducing increased levels of estradiol and estrone [22,23]. This same relationship between obesity and decreased T has been observed in animal models [24,25]. Adipose tissue functions as an endocrine organ that secretes adipocytokines, including leptin and adiponectin, as well as inflammatory mediators [20].

Leptin, in addition to stimulating the satiety center, acts centrally and peripherally; centrally it stimulates KISS-1 neurons, which secrete KISS-1 peptide to stimulate GnRH neurons and stimulate LH secretion, thereby stimulating the conversion of 17-hydroxy progesterone to T in Leydig cells [14,26].

Ruiz-Valderrama et al. (2025) [27], in a study performed in rats with similar equal conditions of diet and time of administration, reported a tendency to decrease testosterone levels in animals that were overweight and a significant decrease in the presence of obesity. This effect may be caused at two levels: the first one related to the deregulation and loss of the feedback systems of the hypothalamic–pituitary–gonadal axis, and the second one is at the level of steroidogenesis.

It has been reported that the administration of a high-fat diet in rats [28] alters steroidogenesis in Leydig cells by suppressing the expression of genes such as StAR and CYP 11 A (limiting step in steroid biosynthesis), as well as in the transcription of genes of the enzymes that metabolize pregnenolone to testosterone; on the other hand, in Sertoli cells, a decrease of androgen receptors is observed, affecting spermatogenesis.

In the present study, we observed histological changes at the tubular level in the testis, which are more evident in obese animals. The most visible changes are the folding of the basement membrane, detachment of the seminiferous epithelium, as well as a certain disarray in spermatogenesis; these changes may be related to the probable decrease in testosterone. In another study using the same food to generate overweight and obese rats, the decrease in testosterone levels was inversely proportional to the males’ weight [29].

It is known that adequate levels of leptin are required both at a testicular level and in neural circuits to maintain the activity of the hypothalamus–pituitary–testes axis. Increased leptin induces a “leptin resistance” response that may ultimately lead to functional hypogonadism due to testosterone deficiency. In addition, adipose tissue is directly involved in the decrease of circulating androgens by the conversion of testosterone to o-estradiol, which in turn acts indirectly on T synthesis at the hypothalamic level [30].

In the overweight and obese groups, in addition to disordered seminiferous epithelium, a lower presence of spermatozoa in the lumen of the tubules can be observed. Tena-Sempere et al. (2002) reported that leptin can cross the blood–testicular barrier to modulate steroidogenic processes. In the gonad, leptin receptors are found only in Leydig cells, and it has been reported that leptin levels are inversely correlated with testosterone concentration, inducing abnormalities in sperm production [31].

Another androgen-dependent organ is the epididymis, as it requires the presence of androgens to maintain its structure and function [8]; this is a highly specialized organ that synthesizes proteins, glycoproteins, glycolipids, and phospholipids, which are released into the tubule lumen as they are necessary for sperm maturation and survival [8,32].

Overweight/obesity can generate changes in this organ; as fat accumulation at the scrotal level generates an increase in temperature, it has been reported that mainly the cauda region is required to maintain a lower temperature, even lower than the rest of the epididymis and testis, so not to damage the spermatozoa during the storage process [33]. On the other hand, it has been reported that obesity can generate oxidative stress [34], which can alter epididymal morphophysiology, generating cell damage, and consequently, alterations in sperm maturation and storage parameters [35,36]. However, it was reported that the damage caused by overweight and obesity in the epididymal sperm maturation process would be caused mainly by the decrease in T and not by oxidative stress, as observed by Ruíz-Valderrama et al. (2022) [27].

The way in which obesity can cause systemic oxidative stress is by an impairment in the nuclear maturation, resulting in excessive protamine disulfide bridges, abasic sites (which in themselves do not constitute a DNA break), cross-linking of nuclear proteins, and finally DNA breaks (only when oxidative stress is severe) [37,38,39]. Previously, rats with overweight and obesity have been shown to exhibit increases in enzymatic activity, specifically superoxide dismutase (SOD), catalase (CAT), and glutathione peroxidase (GPX), in the testis and epididymis. This increase is sufficient to counteract the oxidative stress environment without damaging the integrity of the DNA [36].

Another cause is hormonal changes. Several authors have reported that the decrease in T causes changes in the epididymal epithelium, such as loss of cilia, accumulation of lysosomes, and the presence of vacuoles with endocytic activity. The changes observed in the histological and morphometric evaluation indicate a decrease in the levels of T. Another consequence of the decrease in T is that it favors the release of pro-apoptotic factors [40], which could be causing the results obtained in the present study.

Even though a qualitative decrease in cilia was observed, it is worth noting that one important difference was the length of the cilia, which was affected in overweight, and to a greater extent in obesity. The presence of these cilia is important for both the regulation and control of the microenvironment and is essential for the epididymal function [41]. The cilia present in the epididymis are non-motile cilia [42,43], so they respond to the extracellular microenvironment using chemical stimuli [42,43].

In the epididymis of overweight animals, the increase in the length of the cilia in both the corpus and cauda is notable in contrast to obese animals, in which they are practically absent. In a study carried out in castrated *Macacus fustatus*, Murakami et al. (1976) [44] reported a correlation between the presence of cilia in the epididymis and testosterone levels; they observed that 15 days post-castration, the cilia were shorter and showed a disarrangement; at 30 days, a regression of stereocilia was observed; these effects were reversible with the administration of testosterone. Obese animals show similar characteristics to those of castration effects.

In our study model, the animals are subjected to a condition that not only can affect hormone levels but can also generate an inflammatory process and changes in the epididymal fluid that can be detected by the cilia involved in the maintenance of tissue homeostasis in adults. It is likely that in this process, the Hedgehog signaling pathway responsible for tissue maintenance is affected, with a decrease in the height of the epithelium being observed, as well as an initial change in the length of the cilia as an adaptive mechanism [45,46]. Various ciliopathies have been associated with male fertility problems [41], so it is likely that changes in the structure and/or function of these organelles alter fertility in overweight or obese males.

At the level of the epithelium, in addition to changes in the cilia, vacuoles are also observed in the principal cells. It has been reported that these cells secrete epididymosomes, transport proteins that favor the transport of molecules necessary for sperm maturation, as well as immobilin, a protein that promotes the maintenance of sperm quiescence, so that changes in the function of these cells can alter both function and maturation [44] and sperm motility [14,44,45,46].

On the other hand, clear cells are involved in the process of acidification of the luminal fluid and endocytosis of proteins [8,47,48]; as the pH of the luminal fluid is modified mainly in the cauda region, spermatozoa may become motile during the storage process, therefore, changes in the pH of the luminal fluid will modify the motility and fertilization capacity of the sperm.

In addition to T, o-estradiol is important for the normal function of this organ. Both hormones regulate the onset of sperm motility and the remodeling of the sperm membrane, mainly in the caput and corpus regions [46]. Under conditions of overweight and obesity, the balance between these hormones is affected as there is an increased aromatization of androgens. Both narrow, apical, and clear cells have estrogen receptors (ERs), and it has been reported that an increase in estrogen levels alters the function and physiology of clear cells [49].

The sum of the aforementioned alterations causes changes in sperm quality, drastically reducing sperm vitality (32%) in obese animals and significantly in overweight animals (70%). The presence of malformations in the angulated midpiece, angulated flagellum, and coiled flagellum is also increased in these groups. These alterations can occur due to changes induced in the epididymis, as their morphology and function are altered, modifying the physiological conditions required by the spermatozoa for their maturation and viability. Even in other studies, it has been reported that some apoptosis markers, such as exposure to phosphatidylserine, have increased, which also reaffirms that all the changes mentioned in the epididymis as androgen-dependent organs affect seminal quality [20,27,50].

Although there are morphological alterations in both the testis and the epididymis, it seems that the latter is more sensitive to changes induced by an increase in temperature and hormonal changes induced by overweight and obesity; changes are observed in the somatic epididymal index, in the histology of the epididymal tubule, morphometric parameters, and size of the cilia, which result in a decrease in sperm vitality and an increase in the number of spermatozoa with malformations. Alves et al. (2016) [51] mention that leptin, ghrelin, and glucagon-like peptide-1 are pharmacologically relevant, since these hormones regulate glucose homeostasis in the body and may be ideal targets for anti-obesity therapy. One of the next perspectives is to determine the concentrations of leptin and other hormones in the issue of male infertility and the mechanisms that could be involved, since their potential effect on male reproduction is highly dependent on metabolic cooperation between testicular cells. This remains a matter of debate, and this study seems to shed light on the pharmacological relevance to counteract the infertility problems experienced by overweight and obese men.

## 4. Materials and Methods

### 4.1. Animals and Treatment

Eighteen male rats of the Wistar strain were used, with an age of 3 months and an approximate weight of 300 g, divided into three groups with an equal number of animals (*n* = 6). The rats were obtained from the Vivarium of the Universidad Autónoma Metropolitana– Iztapalapa (UAM-I). The animals were kept following the stipulations of the guidelines approved by the Institutional Committee for the Care and Use of Animals of the UAM Vivarium and the “Regulation of the General Health Law on Health Research” of the Mexican Ministry of Health (NOM-062-ZOO-1999). Water and food were provided ad libitum (each group received a different diet). For 30 consecutive days, feed consumption was recorded, as well as the weight of the animals.

The following inclusion and exclusion criteria were determined: age: 2–9 months, adult male; standard weight: 250–300 g; health condition: healthy. Exclusion criteria: all those who do not meet the above criteria.

Control: standard diet. Purina Rat Chow 5001 food (3% fat). Diet to feed rats with an approximate weight of 300 to 420 g.

Diet Induced Overweight (DIO): Test Diet brand (58Y2/D12450B), 10% fat content (Energy F/fat PD Yellow) [16]. Diet to obtain a weight between 430 and 510 g.

Diet Induced Obesity (DIOb): Test Diet brand (58Y1/D12492), 60% fat content (Energy F/fat PD Blue) [16]. Diet to obtain a weight between 530 and 600 g.

DIO and DIOb diets are composed of saturated fatty acids, monosaturated fatty acids, polyunsaturated fatty acids, and are balanced in percentages and parts per million of linoleic acid, arachidonic acid, Omega-3, and cholesterol.

### 4.2. Lee Index

This index is a morphometric parameter that relates weight and body length; it is considered a predictor of obesity [52]. Lee index = cube root of body weight (g)/naso-anal length (cm); it is considered that the value to determine normal weight is less than 0.3.

The amount of scrotal fat was weighed, and the average per group was obtained.

### 4.3. Gonadosomatic Index (GSI) and Epididymal Somatic Index (ESI)

GSI was calculated by adding the weight of both testes as a proportion of the total body weight, and ESI was calculated by adding the weight of both epididymides as a proportion of the total body weight. In both cases, the somatic index represents the percentage of body mass allocated to the testis and epididymis.

### 4.4. Sample Collection

The animals were slaughtered by decapitation (NOM-062-ZOO-1999). An incision was made at the ventral level from the terminal region of the sternum to the pubic symphysis, the fat surrounding the gonads was separated, and both testicles and epididymis were removed. The somatic epididymal index was weighed and calculated, as well as the somatic testicular index (somatic index = organ weight/body weight × 100%). Both testes, as well as the right epididymis, were fixed in 10% neutral formalin (Formaldehído Mallinckrodt Chemical 5016-08, Mallinckrodt Pharmaceuticals, Dublin, Ireland) for histological analysis, then the routine histological method was followed, and both testicles and epididymis were included in paraffin. The left epididymis was sectioned into its three anatomical regions (*caput*, *corpus*, and *cauda*).

### 4.5. Histological Analysis

- Testicle

First, 4 μm cross-sections were made, then stained with the Hematoxylin–Eosin technique (J.T. Baker 3873, Avantor Performance Materials, LLC, Radnor, PA, USA; Hycel de México 300, Hycel de México, S.A. de C.V., Zapopan, Mexico). Next, 5 slices of each testicle (right and left) were analyzed for quantitative and qualitative analysis; a total of 50 tubules were analyzed; those with a circular shape were considered for histomorphometry, and the parameters analyzed were tubule area, diameter, and height of the seminiferous epithelium.

- Epididymis

First, 4 μm sections were made, then stained with the Hematoxylin–Eosin (H-E), Masson’s Trichromic (TM) technique (Rojo Ponceau Sigma P2395, Sigma-Aldrich, St. Louis, MO, USA; orange G Sigma 0.1625, Sigma-Aldrich, St. Louis, MO, USA; Azul de Anilina Hycel de México 228, Hycel de México, S.A. de C.V., Zapopan, Mexico). Subsequently, qualitative and morphometric analysis of each area of the epididymis was performed.

Qualitative analysis: 20 ducts were analyzed for each region of the epididymis. For the histological description, ductal shape, epithelial type, epithelial changes, and sperm distribution in the duct lumen were considered.

Histomorphometry: Measurements were made in 20 ducts in each region. Duct area, percentage of duct light, percentage of the area of the ducts occupied by sperm, epithelial height, and cilia length were determined. The randomization process consisted of determining, during measurements, the selection of tubules that met a shape factor close to 1, which indicates a circle. The sections were made in series, and sections were analyzed at 100 mm intervals; that is, one section was analyzed, and the next after 25 sections. In this study, this was performed without knowing which treatment the sections analyzed belonged to.

### 4.6. Sperm Analysis

Spermatozoa were extracted in each sectioned epididymal region [53]. Subsequently, the viability and morphology of the spermatozoa were determined following the method described in the Laboratory Manual for the examination and processing of human semen [54] and the manual for the analysis of copulation of the ejaculate of the albino rat (*Rattus norvegicus*) [55]; 200 spermatozoa were analyzed with the sperm vitality test, and eosin nigrosin staining was performed; sperm that were not stained were considered alive, and those that were stained pink were considered dead. In sperm morphology, the number of spermatozoa that presented abnormalities in the head, middle piece, and flagellum, as well as the presence of cytoplasmic droplets, was quantified. The results of both analyses were expressed as a percentage.

### 4.7. Statistical Analysis

Means ± standard deviation (SD) were calculated. To determine statistical differences in the analyzed results, a multiple ANOVA was followed by Tukey’s post hoc test with normal distribution and homogeneous variance. ^a,b,c^ indicate significant differences *p* < 0.01, and ^d,e,f^ *p* < 0.001. The statistical analyses were performed using NCSS 2007 DATA Software, version 1, Inc. (Kaysville, UT, USA).

## 5. Conclusions

Overweight and obesity induce morphological changes in the testis and epididymis, which are also manifested in noticeable changes in spermatobioscopy. The observed changes are induced by hormonal changes that modify the reproductive axis, but also by alterations that may occur at the testicular and epididymal maturation level related to steroid biosynthesis. The results obtained lead us to suggest studies in which testosterone and/or hypolipidics are administered to restore fertility.

## Figures and Tables

**Table 1:** Somatometric parameters: Lee index, scrotal fat, and somatic indices. Multiple ANOVA, Tukey post hoc test. Results are shown as the mean ± SD. ^a,b,c^ indicate significant differences between groups, *p* < 0.01.

	Control	DIO	DIOb
Lee Index (%)	0.291	0.32	0.33
Scrotal fat (g)	3.75 ± 0.87 ^a^	6.61 ± 0.42 ^b^	13.31 ± 4.48 ^c^
GSI (%)	0.94 ± 0.04 ^a^	0.98 ± 0.03 ^a^	0.80 ± 0.03 ^b^
ESI (%)	0.36 ± 0.29 ^a^	0.35 ± 0.027 ^a^	0.80 ± 0.0017 ^b^

**Table 2:** Histomorphometric analysis of the testis. Multiple ANOVA, Tukey post hoc test. Results are shown as the mean ± SD. ^d,e,f^ indicate significant differences between groups (*p* < 0.001).

	Control	DIO	DIOb
Seminiferous tubule diameter (µm)	306.21 ± 33.37 ^d^	310.13 ± 36.82 ^d^	333.23 ± 25.59 ^e^
Height of the seminiferous epithelium (µm)	75.58 ± 14.46 ^d^	66.37 ± 10.07 ^e^	82.93 ± 12.37 ^f^

**Table 3:** Histomorphometric analysis of the epididymis. Multiple ANOVA, Tukey post hoc test. Results are shown as the mean ± SD. ^d,e^ indicate significant differences between groups, *p* < 0.001.

		Control	DIO	DIOb
Duct area (µm^2^)	*Caput*	10,953 ± 540.3 ^d^	22,911 ± 540.3 ^e^	27,172 ± 540.3 ^e^
	*Corpus*	27,866 ± 523.29 ^d^	25,189 ± 523.29 ^e^	21,723 ± 523.29 ^e^
	*Cauda*	28,770.23 ± 489.4 ^d^	24,149.41 ± 489.4 ^e^	25,149 ± 489.4 ^e^
Duct lumen (%)	*Caput*	42.3 ± 0.55 ^d^	66.4 ± 0.55 ^e^	64.8 ± 0.55 ^e^
	*Corpus*	67.4 ± 0.49 ^d^	65.3 ± 0.49 ^e^	67.6 ± 0.02 ^d^
	*Cauda*	70.83 ± 0.27 ^d^	67.71 ± 0.27 ^e^	68.8 ± 0.27 ^e^
Area of the ducts occupied by sperm (%)	*Caput*	15.5 ± 0.46 ^d^	42.2 ± 0.46 ^e^	48.44 ± 0.46 ^e^
	*Corpus*	44.94 ± 0.48	43.87 ± 0.48	44.1 ± 0.48
	*Cauda*	44.05 ± 0.38 ^d^	45.2 ± 0.38 ^d^	52.49 ± 0.38 ^e^
Epithelial height (µm)	*Caput*	20.5 + 0.2 ^d^	15.66 ± 0.13 ^e^	14.55 ± 0.13 ^e^
	*Corpus*	16.9 ± 0.16 ^d^	15.8 ± 0.16 ^e^	15.3 ± 0.16 ^e^
	*Cauda*	13.9 ± 0.13 ^d^	15.8 ± 0.16 ^e^	13.35 ± 0.13 ^d^

**Table 4:** Cilia length. Multiple ANOVA, Tukey post hoc test. Results are shown as the mean ± SD. ^d,e,f^ indicate significant differences between groups, *p* < 0.001.

	Control	DIO	DIOb
*Caput* (µm)	142.24 ± 21.35 ^d^	40.24 ± 21.35 ^e^	10.09 ± 21.35 ^f^
*Corpus* (µm)	52.94 ± 15. 24 ^d^	71.02 ± 15.24 ^e^	15.89 ± 15.24 ^f^
*Cauda* (µm)	21.91 ± 17. 88 ^d^	102.50 ± 17.88 ^e^	55.02 ± 17.88 ^f^

**Table 5:** Spermatobioscopy. Multiple ANOVA, Tukey post hoc test. Results are shown as the mean ± SD. ^d,e,f^ indicate significant differences between groups, *p* < 0.001.

	Control	DIO	DIOb
Vitality (%)	96 ± 3.1 ^d^	70 ± 3.1 ^e^	32 ± 3.1 ^f^
Abnormalities (%)			
Amorphous head	1 ± 0.2	2 ± 0.2	3 ± 0.2
Middle piece			
angulated	7 ± 1.9 ^d^	19 ± 1.9 ^e^	34 ± 1.9 ^f^
and asymmetric	0	1 ± 1.02	1 ± 0.2
Flagellum			
Angled	13 ± 3 ^d^	45 ± 3 ^e^	52 ± 3 ^f^
Coiled	2 ± 3.4 ^d^	14 ± 3.4 ^e^	23 ± 3.4 ^f^

## Data Availability

The dataset is available on request from the authors.

## Abbreviations

The following abbreviations are used in this manuscript:

DNA	Desoxyribonucleic Acid
E2	Estradiol
E-N	Eosin Nigrosine
ER	Estrogen Receptors
FSH	Follicle-Stimulating Hormone
GNRH	Hypothalamic Gonadotropin-Releasing Hormone
H-E	Hematoxylin–Eosin
KISS-1	metastasis suppressor gene
LH	Luteinizing Hormone
SPI	Epidydimal Somatic Index
STI	Somatic testicular index
T	Testosterone
MT	Masson’s Trichromic
WHO	World Health Organization
